# Response of Calcium-Dependent Protein Kinase Genes’ Expression in ‘Feizixiao’ Litchi Pulp to Foliar Nutrient Treatment of Calcium–Magnesium Mixed Solution and Their Regulation of Sugar Transformation

**DOI:** 10.3390/plants14111583

**Published:** 2025-05-23

**Authors:** Jiabing Jiao, Ling Wei, Shaopu Shi, Yijia Gao, Chenyu Jiang, Muhammad Sajjad, Kaibing Zhou

**Affiliations:** 1 Sanya Institute of Breeding and Multiplication, Hainan University, Sanya 572025, China; 23210902000009@hainanu.edu.cn (J.J.); 23110901000046@hainanu.edu.cn (L.W.); 22210902000016@hainanu.edu.cn (S.S.); 23220951310127@hainanu.edu.cn (Y.G.); 23220951310128@hainanu.edu.cn (C.J.); drmuhammadsajjad@hainanu.edu.cn (M.S.); 2 Key Laboratory of Quality Regulation of Tropical Horticultural Crop in Hainan Province, School of Tropical Agriculture and Forestry, Hainan University, Haikou 570228, China

**Keywords:** ‘Feizixiao’ litchi, foliar nutrient treatment of calcium–magnesium mixed solution, calcium-dependent protein kinase, gene expression, glycotransformation

## Abstract

Previous studies have shown that foliar spraying with a 0.3% CaCl_2_ + 0.3% MgCl_2_ solution can mitigate the “sugar receding” phenomenon in fruit pulp, partly by regulating sugar conversion in the pulp of ‘Feizixiao’ litchi (*Litchi chinensis* Sonn.). Given that calcium-dependent protein kinases (CDPKs) in plants regulate sugar metabolism by modulating the activity of key sugar conversion enzymes, this study investigated the expression response of *CDPK* genes in ‘Feizixiao’ litchi pulp to foliar calcium–magnesium nutrient treatment and their regulatory characteristics on sugar conversion. After the fruit set, ‘Feizixiao’ litchi trees were subjected to three consecutive foliar spray applications of 0.3% CaCl_2_ + 0.3% MgCl_2_, with water spraying as the control. The dynamic changes in peel *h* values and soluble sugar and monosaccharides, water-soluble calcium (Ca^2+^) and magnesium (Mg^2+^), plant hormones, and the concentration of CDPKs in the pulp were compared throughout fruit development. Key differentially expressed members of the *CDPK* gene family were screened through real-time quantitative PCR analysis. The results showed that the peel color transition occurred earlier in the control (CK) than in the treatment (T), but the coloration process accelerated in the treated fruit, leading to no significant difference in peel *h* values between the groups at 76 days after anthesis (DAA), when both reached the lowest levels. The total of soluble sugar in the pulp peaked at 70 DAA in both groups, but while the CK exhibited a significant decline thereafter, T maintained stable sugar levels, thereby mitigating the “sugar receding” phenomenon. Water-soluble calcium and water magnesium levels were significantly higher in the T at 42 and 63 DAA, with water calcium remaining significantly higher at 70 DAA. Furthermore, sucrose, glucose, fructose, abscisic acid (ABA) contents, and CDPK concentration were significantly higher in the T at 70 and 76 DAA. The *CDPK* gene family members *LcCDPK1*, *LcCDPK2*, *LcCDPK3*, *LcCDPK4*, *LcCDPK5*, *LcCDPK9*, *LcCDPK15*, and *LcCDPK17* were upregulated in response to T. Among them, *LcCDPK1*, *LcCDPK4*, *LcCDPK5*, *LcCDPK9*, and *LcCDPK17* were identified as key structural genes due to their significant correlation with soluble sugar content and CDPK concentration, as well as their differential expression between T and CK. In conclusion, foliar calcium–magnesium nutrient treatment upregulates the expression of these five CDPK gene family members by increasing the ABA levels in the pulp, leading to more CDPK accumulation. This accumulation inhibits sugar conversion and promotes sucrose and fructose accumulation, thereby mitigating the “sugar receding” phenomenon in ‘Feizixiao’ litchi pulp.

## 1. Introduction

Litchi (*Litchi chinensis* Sonn.), a tropical fruit tree belonging to the Sapindaceae family, is mainly distributed in the Guangdong, Guangxi, Fujian, Hainan, and Taiwan provinces, and is the largest cultivated fruit tree in South China [1]. The ‘Feizixiao’ litchi is one of the main varieties in litchi producing areas in China. Its pulp is aromatic, tender, sweet, and juicy, and it is an excellent variety with good flavor. Moreover, the fruit peel exhibits a phenomenon known as “delayed degreening”, in which its coloration lags behind the increase in fruit flesh sugar content, preventing full red pigmentation on the fruit surface. Conversely, when the fruit surface attains full redness, the sugar content in the fruit flesh declines, leading to a deterioration in flavor and increased acidity. This phenomenon is referred to as “sugar receding” in the fruit flesh [2,3]. This severely affects its commercial value as a fresh fruit. Foliar nutrient treatment of calcium–magnesium mixed solution could effectively mitigate the “sugar receding” phenomenon in the pulp of ‘Feizixiao’ litchi [4,5]. One of the underlying mechanisms was found to be that this treatment inhibited aerobic respiration via the Embden–Meyerhof–Parnas-tricarboxylic acid (EMP-TCA) and pentose phosphate pathway (PPP), while promoting the cytochrome respiration pathway (CP) and suppressing cyanide-resistant respiratory pathways (AP) [6,7]. In terms of sugar metabolism, the treatment inhibited hexokinase (HK) gene expression, and thereby suppressed HK activity and subsequently inhibited glucose glycolysis. This regulation helped maintain a relatively high dynamic balance of sugar conversion in the pulp, effectively mitigating the “sugar receding” phenomenon [5]. However, its signal transduction mechanism remains unknown.

As a non-saltation fruit, abscisic acid (ABA) plays a key role in the regulation of pulp ripening and development [8]. Ca^2+^ plays a crucial role in ABA signal transduction [9,10], with one of its signaling pathways involving CDPK, which participates in various physiological processes in plants [11].

The CDPK is a plant-specific serine/threonine (Ser/Thr) protein kinase that contains a calmodulin-like (CAM) domain in its structure. It can sense intracellular Ca^2+^ signals and regulate downstream target proteins through phosphorylation [12,13]. CDPKs in plants are typically encoded by a large multigene family, with potential functional redundancy and/or diversity. As a result, they are considered an essential component of the plant hormone signal transduction network [14]. ABA could induce the activity of a class of CDPKs, which sense intracellular Ca^2+^ signals and regulate sugar metabolism by phosphorylating downstream substrates [12,13]. Studies on the physiological functions of CDPKs have also shown that they could regulate the activity of key enzymes involved in sugar metabolism [15]. For example, CDPKs could promote the phosphorylation of sucrose-phosphate synthase (SPS) at Ser424, thereby activating SPS and increasing the intracellular sucrose content [16].

Based on the aforementioned ABA signal transduction and physiological functions of CDPKs, it is speculated that pulp ABA signaling and CDPKs may be involved in the regulation of sugar conversion under the foliar nutrient treatment of calcium–magnesium mixed solution. Therefore, this study preliminarily explores the expression characteristics of *CDPK* genes in pulp in response to the foliar nutrient treatment and investigates their signal transduction mechanism in sugar conversion regulation.

## 2. Results

### 2.1. Effects of the Treatment on Peel Coloration

**Figure 1:**
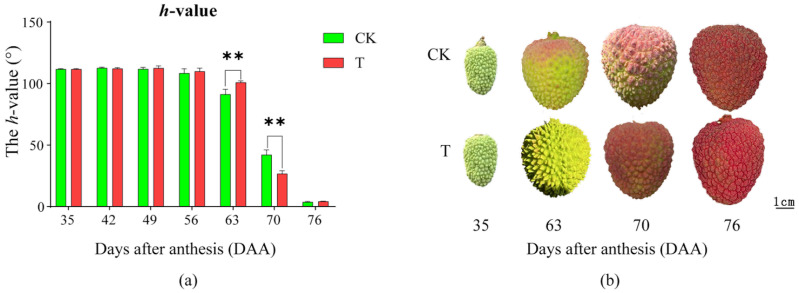
The effects of 0.3% CaCl_2_ + 0.3% MgCl_2_ treatment on peel coloration. (**a**) *h* value. (**b**) Phenotypic maps of litchi 35, 63, 70, and 76 DAA. A *t*-test was used to assess the significance of differences between T and CK at the same sampling time. n = 3. ** indicates *p* < 0.01; the error line is expressed by standard error.

The effect of the treatment on peel coloration is shown in Figure 1a,b. Before 56 DAA, no significant changes were observed in either T or CK, with the peel remaining green. At 63 DAA, the *h* value of both T and CK began to decline, indicating the onset of peel reddening, which continued thereafter. At 63 DAA, CK showed a significantly lower *h* value than T, suggesting that color transition occurred earlier in CK. By 70 DAA, CK exhibited a significantly higher *h* value than T, indicating that T accelerated coloration, leading to a redder peel than CK. At 76 DAA, there was no significant difference between T and CK, suggesting that during this period, both had fully turned red. In summary, T delayed the onset of peel coloration but accelerated the coloration process, resulting in a redder peel than CK one week before full coloration, while there was no difference in peel color at full maturation.

### 2.2. Effects of the Treatment on the Total of Soluble Sugar and the Contents of Sugar Components in Pulp

**Figure 2:**
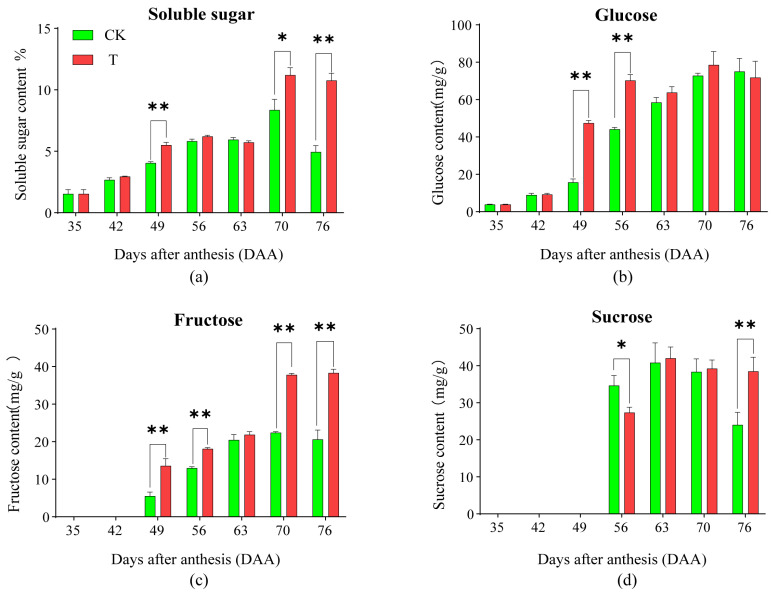
The dynamic change of soluble sugar and sugar components in pulp between CK and T. (**a**) Soluble sugar content. (**b**) Glucose content. (**c**) Fructose content. (**d**) Sucrose content. A *t*-test was used to assess the significance of differences between T and CK at the same sampling time. n = 3. * indicates *p* < 0.05; ** indicates *p* < 0.01; the error line is expressed by standard error.

The effects of the treatment on the dynamic changes in the total of pulp soluble sugar and the contents of the monosaccharide in pulp are shown in Figure 2a–d. For soluble sugar content, both T and CK exhibited an overall increasing trend, reaching a peak at 70 DAA. However, CK showed a decline at 76 DAA, leading to the occurrence of the “sugar receding” phenomenon, while T maintained peak levels at 76 DAA, thereby preventing “sugar receding”. At 49, 70, and 76 DAA, T was significantly higher than CK. For glucose content, CK showed a continuous increasing trend and reached its peak at 76 DAA. T exhibited a rapid increase during the early period, showed no significant change at 63 DAA, reached its peak at 70 DAA, and remained stable at 76 DAA. T was significantly higher than CK at 49 and 56 DAA, while no significant differences were observed at other sampling times. For fructose content, both T and CK showed an overall increasing trend, reaching the peak at 70 DAA and maintaining the highest level at 76 DAA. However, the peak value in T was twice as high as that of CK. Fructose was undetectable at 35 and 42 DAA. At 49, 56, 70, and 76 DAA, T was significantly higher than CK, with no significant differences observed between the two at other sampling times. For sucrose content, both T and CK exhibited an inverted “V-shaped” trend, increasing initially and then declining, with peak values at 63 DAA. Afterward, CK showed a sharp decline, whereas T showed a more gradual decrease. Sucrose was undetectable before 49 DAA. At 56 DAA, CK was significantly higher than T, whereas at 76 DAA, T was significantly higher than CK, with no significant differences observed between the two at other sampling times. Thus, the significantly higher soluble sugar content in T at 49 DAA was due to increasing glucose and fructose accumulation. This might be due to the previous processing that accumulated up to 49 days, resulting in this outcome. Fructose accumulation increased at 70 DAA, and fructose and sucrose accumulation increased at 76 DAA. These results indicate that the treatment mitigated “sugar receding” by promoting the accumulation of sucrose and fructose in the pulp.

### 2.3. Effects of the Treatment on Water-Soluble Calcium and Magnesium in Pulp

**Figure 3:**
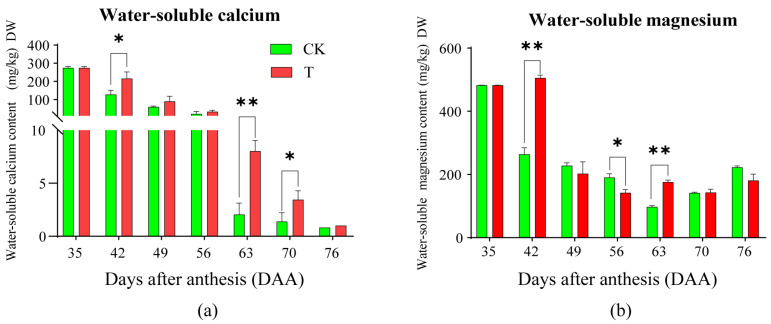
The dynamic change of water-soluble calcium content (**a**) and water-soluble magnesium content (**b**) in pulp between CK and T. A *t*-test was used to assess the significance of differences between T and CK at the same sampling time. n = 3. * indicates *p* < 0.05; ** indicates *p* < 0.01; the error line is expressed by standard error.

The effect of the treatment on the dynamic changes in water-soluble calcium content in pulp are shown in Figure 3a. Both T and CK exhibited a decreasing trend after anthesis, with T declining more slowly than CK, and both reaching their lowest values at 76 DAA. At 42, 63, and 70 DAA, T was significantly higher than CK, while no significant differences were observed between the two at other sampling times. The effect of the treatment on the dynamic changes in water-soluble magnesium content in the pulp are shown in Figure 3b. CK exhibited a “V-shaped” trend, first decreasing and then increasing, reaching its lowest value at 63 DAA before rising. In T, no significant change was observed at 35 and 42 DAA, followed by a sharp decline, reaching the lowest value at 56 DAA, and remaining stable thereafter. T was significantly higher than CK at 42 and 63 DAA, while CK was significantly higher than T at 56 DAA, with no significant differences observed between the two at other sampling times. These results indicate that the treatment significantly affected water-soluble calcium and magnesium levels in the pulp, particularly after the first field application and during the later growth stages. Combined with the dynamic changes in soluble sugar content, these findings suggest that T enhances sugar accumulation by increasing water-soluble calcium and magnesium levels in the pulp.

### 2.4. Effects of the Treatment on the Content of Seven Plant Hormones in Pulp

**Figure 4:**
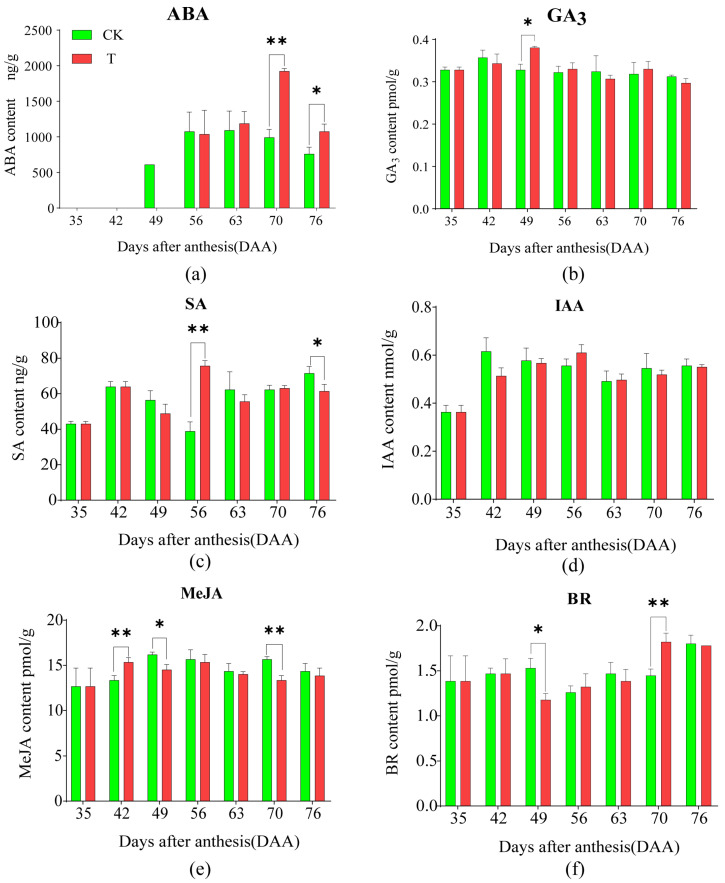
The effects of 0.3% CaCl_2_ + 0.3% MgCl_2_ treatment on seven plant hormones in pulp. (**a**) ABA content. (**b**) GA_3_ content. (**c**) SA content. (**d**) IAA content. (**e**) MeJA content. (**f**) BR content. A *t*-test was used to assess the significance of differences between T and CK at the same sampling time. n = 3. * indicates *p* < 0.05; ** indicates *p* < 0.01; the error line is expressed by standard error.

The effects of the treatment on the dynamic changes in ABA content in pulp are shown in Figure 4a. Both T and CK exhibited an inverted “V-shaped” trend, with CK peaking at 63 DAA and T peaking at 70 DAA, followed by a decline. ABA was nearly undetectable at 35 and 42 DAA. At 70 and 76 DAA, ABA content in T was significantly higher than in CK, with no significant differences observed between the two at other sampling times.

The effects of the treatment on GA_3_ content in pulp are shown in Figure 4b. No significant changes were observed in either T or CK throughout fruit development, except at 49 DAA, when T was significantly higher than CK.

The effects of the treatment on SA content in pulp are shown in Figure 4c. CK exhibited an initial increase, followed by a decline and a subsequent increase, with the highest peak at 76 DAA and the lowest at 56 DAA. T showed a highly significant fluctuation, peaking at 56 DAA and reaching its lowest point at 35 DAA. At 56 DAA, SA content in T was significantly higher than in CK, while at 76 DAA, it was significantly lower, with no significant differences observed between the two at other sampling times.

The effects of the treatment on IAA content in pulp are shown in Figure 4d. Both T and CK exhibited an “inverted L-shaped” trend, with a significant initial increase followed by a stable phase. No significant differences were observed between T and CK at any sampling times.

The effects of the treatment on MeJA content in pulp are shown in Figure 4e. Both T and CK exhibited an “M-shaped” fluctuation. At 42 DAA, T was significantly higher than CK, while at 49 and 70 DAA, T was significantly lower than CK, with no significant differences observed between the two at other sampling times.

The effects of the treatment on BR content in pulp are shown in Figure 4f. Both T and CK exhibited a “V-shaped” fluctuation. At 49 DAA, T was significantly lower than CK, while at 70 DAA, T was significantly higher than CK.

**Figure 5:**
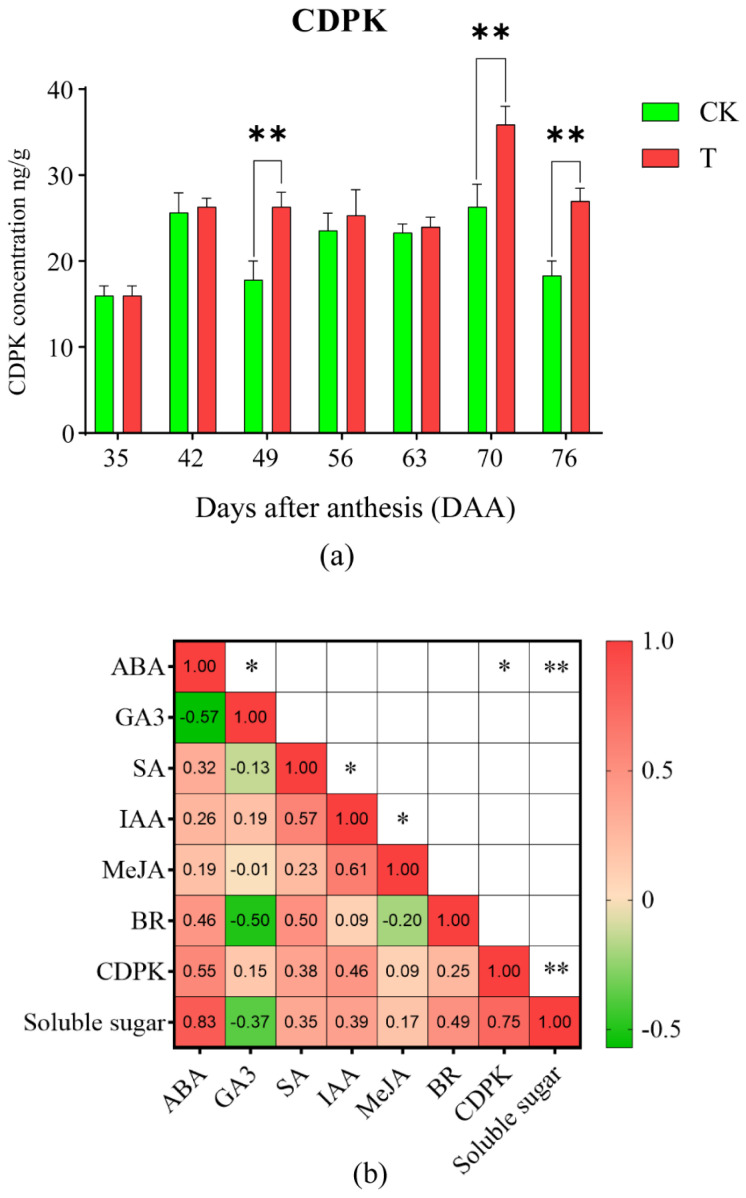
The dynamic change of calcium-dependent protein kinase (CDPK) concentration (**a**) and its correlation analysis with seven plant hormones. A *t*-test was used to assess the significance of differences between T and CK at the same sampling time. n = 3. ** indicates *p* < 0.01; the error line is expressed by standard error. (**b**) In pulp between CK and T. Values indicate the Pearson correlation coefficient; * indicates *p* < 0.05; ** indicates *p* < 0.01.

The results of the multiple linear correlation analysis between soluble sugar content, plant hormones, and CDPK levels in the pulp are shown in Figure 5b. At 70 and 76 DAA, ABA content in T was higher than in CK, showing a highly significant positive correlation with soluble sugar content (r = 0.83, *p* = 0.0002). Other plant hormones showed no significant correlation with soluble sugar content. These findings suggest that the treatment’s mitigation of the “sugar receding” phenomenon of ‘Feizixiao’ litchi pulp may be via increasing ABA levels.

### 2.5. Effects of the Treatment on the Concentration of CDPK in Litchi Pulp

The effects of the treatment on the dynamic changes in CDPK concentration in pulp are shown in Figure 5a. Both T and CK exhibited an “M-shaped” trend, with peaks appearing at 70 DAA. CDPK concentration in T was significantly higher than in CK at 49, 70, and 76 DAA, while no significant differences were observed between the two at other sampling times. A comprehensive analysis incorporating the results from Figure 5b revealed a significant positive correlation between CDPK concentration and soluble sugar content (r = 0.75, *p* = 0.0021), suggesting that T may increase CDPK accumulation, which in turn enhanced the soluble sugar content, ultimately mitigating the “sugar receding” phenomenon of ‘Feizixiao’ litchi pulp. Furthermore, Figure 5b also demonstrates a significant positive correlation between ABA content and CDPK concentration (r = 0.55, *p* = 0.0403), indicating that ABA may positively regulate CDPK accumulation in the pulp.

### 2.6. ‘Feizixiao’ Litchi CDPK Gene Family RT-qPCR Analysis

**Figure 6:**
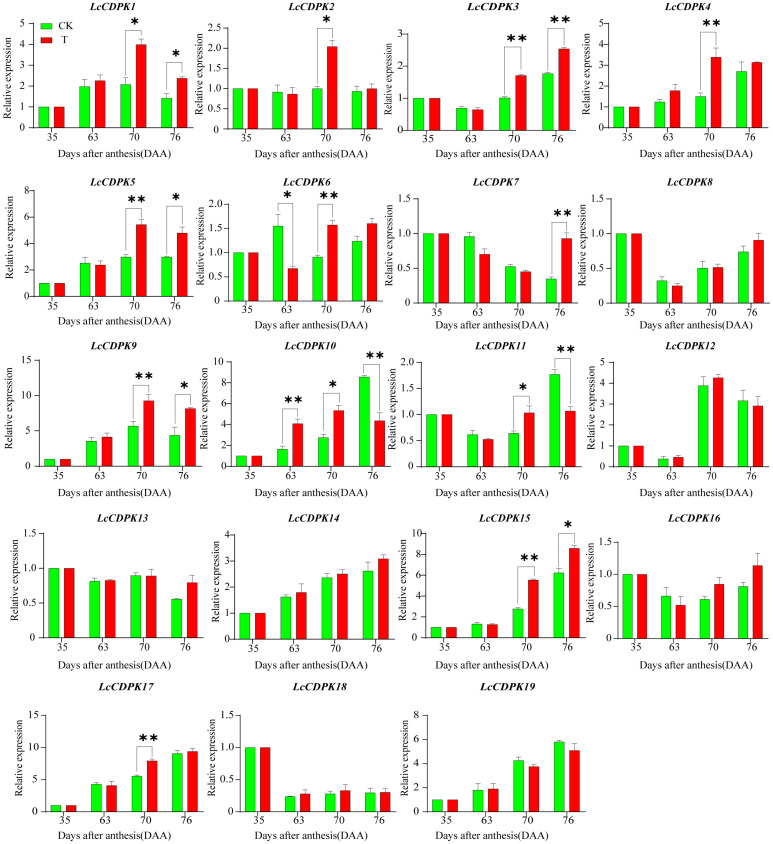
Real-time fluorescence quantification of the *CDPK* gene family in ‘Fezixiao’ litchi. A *t*-test was used to assess the significance of differences between T and CK at the same sampling time. * indicates *p* < 0.05; ** indicates *p* < 0.01; the error line is expressed by standard error.

The results of the RT-*q*PCR analysis of nineteen structural genes in the pulp *CDPK* gene family are shown in Figure 6. In T, *LcCDPK6* was expressed at 63 DAA and was significantly downregulated. At 70 DAA, 11 members—*LcCDPK1*, *LcCDPK2*, *LcCDPK3*, *LcCDPK4*, *LcCDPK5*, *LcCDPK6*, *LcCDPK9*, *LcCDPK10*, *LcCDPK11*, *LcCDPK15*, and *LcCDPK17*—were upregulated. At 76 DAA, *LcCDPK1*, *LcCDPK3*, *LcCDPK5*, *LcCDPK9*, and *LcCDPK15* remained upregulated, whereas *LcCDPK2*, *LcCDPK4*, *LcCDPK6*, and *LcCDPK17* showed no significant difference from CK, and *LcCDPK10* and *LcCDPK11* were significantly downregulated. These findings indicate that the expression trends of *LcCDPK1*, *LcCDPK2*, *LcCDPK3*, *LcCDPK4*, *LcCDPK5*, *LcCDPK9*, *LcCDPK15*, and *LcCDPK17* were consistent with the dynamic changes in soluble sugar and CDPK concentration.

**Figure 7:**
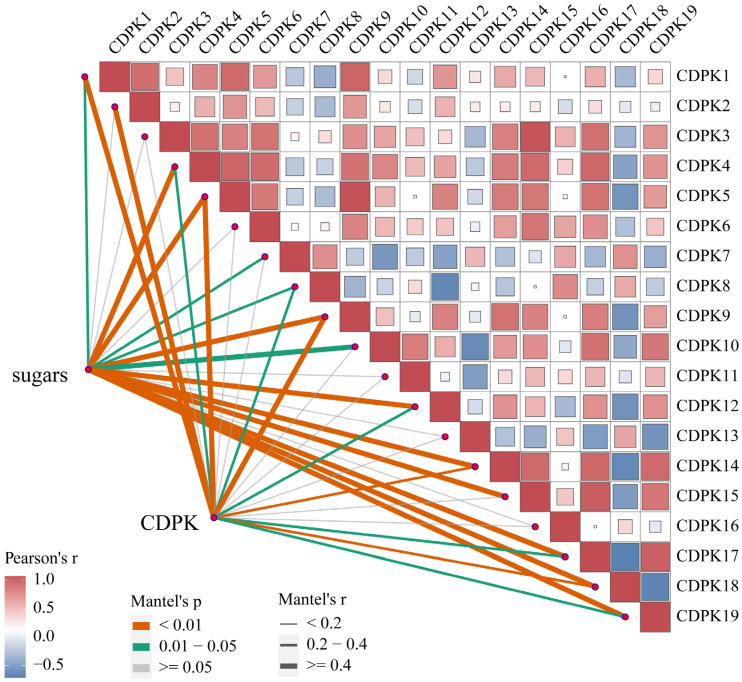
Correlation analysis among soluble sugar, CDPK content, and the *CDPK* gene family.

The results of a correlation analysis between soluble sugar content, CDPK concentration, and the expression levels of *LcCDPK* gene family members are shown in Figure 7. The significantly correlated genes included *LcCDPK1*, *LcCDPK4*, *LcCDPK5*, *LcCDPK8*, *LcCDPK9*, *LcCDPK12*, *LcCDPK14*, *LcCDPK17*, *LcCDPK18*, and *LcCDPK19*. However, the expression levels of *LcCDPK8*, *LcCDPK12*, *LcCDPK14*, *LcCDPK18*, and *LcCDPK19* showed no significant differences at 70 and 76 DAA, indicating that *LcCDPK1*, *LcCDPK4*, *LcCDPK5*, *LcCDPK9*, and *LcCDPK17* were the key differentially expressed genes regulating CDPK accumulation and sugar content. A comprehensive analysis integrating previous results suggests that the treatment increased the ABA content in the pulp, which subsequently upregulated the expression of these five key *LcCDPK* gene family members. This upregulation led to increased CDPK accumulation, promoting a higher level of sugar conversion balance in the pulp, ultimately mitigating the “sugar receding” phenomenon.

## 3. Discussion

### 3.1. The Treatment to Mitigate the ‘Feiziao’ Litchi Pulp “Sugar Receding” Phenomenon

The results of this study indicate that foliar calcium–magnesium nutrition effectively mitigates the “sugar receding” phenomenon in ‘Feizixiao’ litchi pulp, which is consistent with the findings of multiple previous multi-location trials conducted by our research group [6,7,17]. This suggests that the treatment can be applied as a cultivation technique to mitigate the “sugar receding” phenomenon in ‘Feizixiao’ litchi pulp. At 7 days after the first treatment (42 DAA), water-soluble calcium and magnesium contents increased significantly, while sugar and CDPK levels showed no significant changes. At 7 days after the second treatment (49 DAA), both sugar and CDPK levels exhibited relatively significant changes. This may be attributed to either the cumulative effects of the earlier application or a time lag in the treatment response.

### 3.2. Synergistic Regulation of Pulp Sugar Conversion by Foliar Calcium–Magnesium Nutrition

The results of this study indicate that the treatment significantly increased the contents of water-soluble calcium and magnesium, ABA, fructose, and sucrose in the pulp. Moreover, the ABA content exhibited a highly significant positive correlation with the soluble sugar content, which is consistent with previous reports on other crops. Ca^2+^ and Mg^2+^ synergistically regulated the sugar content in maize kernels by influencing endogenous hormones and antioxidant enzyme activities [18]. Foliar calcium application significantly enhanced the activities of SPS, sucrose synthase (SS), and sorbitol oxidase (SOX) in apple fruits, thereby increasing soluble sugar accumulation [19]. These findings are also supported the hypothesis that ABA activates sugar metabolism enzymes through CDPKs [15,20,21], as ABA could induce the activation of CDPKs, thereby enhancing the synthesis activity of key sugar-converting enzymes and ultimately increasing sucrose accumulation. Ca^2+^ is a known activator of CDPKs [22], while Mg^2+^ serves as an essential cofactor [15]. This suggests that foliar calcium–magnesium nutrition enhances Ca^2+^ and Mg^2+^ levels in the pulp, thereby intensifying the activation of the CDPK signaling pathway. Consequently, this promotes the synthesis activity of sugar-metabolizing enzymes, such as SPS, leading to an increase in the accumulation of sucrose and other soluble sugars [23].

Previous studies demonstrated that abiotic stress elevated Ca^2+^ levels in plant tissues, induced *CDPK* expression, and thereby enhanced stress resistance and disease resistance [14,24]. This process was frequently accompanied by an increase in pulp soluble sugar content [25,26]. The present study further confirms that *CDPK* positively regulates the accumulation of soluble sugars in fruit.

## 4. Materials and Methods

### 4.1. Experimental Materials

In the litchi garden (19.9° N, 109.8° E) of Jinpai Farm, Lingao County, Hainan Province, 10–20-year old ‘Feizixiao’ litchi trees were selected as the sample trees. The orchard is located in a tropical monsoon maritime climate, characterized by abundant sunlight and synchronous rainfall and heat conditions. The annual average temperature ranges from 23 to 24 °C, with no frost throughout the year. The annual precipitation is between 1100 and 1800 mm, and the annual average sunshine duration is 2175 h. The soil is fertile lateritic red soil, in which the total calcium content is relatively low, typically around 0.1~0.5 g/kg, while the total magnesium content ranges from 0.5 to 2.0 g/kg. The flowering period of litchi in this orchard occurs from February to March. The physiological fruit drop phase begins in early April, followed by the fruit enlargement stage in late April. The fruit reaches maturity in mid-May.

### 4.2. Experimental Design, Field Treatment, and Sampling Methods

Ten experimental litchi trees were divided into treatment (T) and control (CK) groups, with a single-tree plot design and five replicates. In the T group, a mixed aqueous solution of 0.3% CaCl_2_ + 0.3% MgCl_2_ was sprayed on both the adaxial and abaxial surfaces of the leaves until runoff, while the CK group was sprayed with an equal volume of distilled water in the same manner. Prior to the first foliar fertilization treatment on April 14 (35 days after anthesis (DAA), when the aril had just fully enveloped the seed), five medium-sized fruits located around the middle section of the outer canopy of each experimental tree were selected as reference fruits for dynamic sampling. These reference fruits were marked with tags, and subsequent samplings were conducted based on fruits at the same developmental stage, selected according to the size and coloration of the reference fruits. Before each foliar fertilization treatment, 30 fruits were randomly and evenly collected from the middle section of the outer canopy of each tree. Foliar calcium–magnesium nutrient treatments were immediately applied after each sampling. Two additional foliar fertilization treatments were conducted on April 21 (42 DAA) and April 28 (49 DAA). Further samplings were carried out on May 5, 12, 19, and 25 (56, 63, 70, and 76 DAA), totaling seven sampling events. After each sampling, fruit peel coloration parameters were measured on-site. The samples were then rapidly frozen in liquid nitrogen and transported to the laboratory for storage at −80 °C in an ultra-low-temperature freezer for further analysis.

### 4.3. Determination Methods of Physiological and Biochemical Indexes

#### 4.3.1. Peel Color

The fruit peel hue angle (*h*) was measured using a portable colorimeter. Four measurement points were randomly selected on the equatorial region of the fruit, with an additional point taken at both the pedicel and the apex. The arithmetic mean of the measurements from these six points was calculated and recorded as the fruit coloration index.

#### 4.3.2. Determination of Sugar Content in Pulps

The determination of soluble sugars followed the method described by Cao Jiankang (2007) [27], while the analysis of individual soluble sugar components was conducted according to the method of Wang Jing et al. (2001) [28]. Precisely 1 g of litchi aril was weighed and subjected to microwave kill-out for 30 s. The sample was homogenized with 5 mL of 90% ethanol and centrifuged at 10,000× *g* for 15 min. The supernatant was collected, and the residue was re-extracted with another 5 mL of 90% ethanol. The supernatants were combined and evaporated to dryness in a 90 °C water bath. The residue was redissolved in water to a final volume of 10 mL. After filtration through a 0.45 μm membrane, the sample was ready for analysis. The analysis was performed using a Waters 2695 high-performance liquid chromatography (HPLC) system (Waters, USA) equipped with an Amide column (250 mm × 4.6 mm). The mobile phase consisted of acetonitrile and water (7:3, *v*/*v*), with a flow rate of 1 mL/min. The column temperature was maintained at 35 °C, and the injection volume was 10 μL. Analytical-grade fructose, sucrose, and glucose were used as standards, and chromatographic-grade acetonitrile was used as the mobile phase.

#### 4.3.3. Water-Soluble Calcium and Magnesium

Precisely 0.1 g of dried sample was weighed and dissolved in deionized water, followed by shaking overnight. The sample was then filtered and brought to a final volume of 25 mL. The elemental analysis was conducted using inductively coupled plasma optical emission spectrometry (ICP-OES).

#### 4.3.4. Determination of Plant Hormones Content and CDPK Concentration

The contents of abscisic acid (ABA), brassinosteroids (BR), salicylic acid (SA), indole-3-acetic acid (IAA), gibberellic acid (GA_3_), methyl jasmonate (MeJA), and calcium-dependent protein kinase (CDPK) were determined using commercial enzyme-linked immunosorbent assay (ELISA) kits (Shanghai Meilian Biotechnology Co., Ltd., Shanghai, China), according to the manufacturer’s instructions. Briefly, 0.1 g of litchi aril was weighed and homogenized with 0.9 mL of phosphate-buffered saline (PBS, pH 7.4). The homogenate was centrifuged at 6000× *g* for 15 min at 4 °C, and the supernatant was collected for subsequent analysis.

The detection principle of the ELISA kits is based on a double-antibody sandwich method. Each microwell was pre-coated with a specific monoclonal antibody against the target phytohormone (e.g., IAA, MeJA, SA, BR, GA_3_, ABA) and CDPK. During the assay, standards or samples, along with horseradish peroxidase (HRP)-conjugated detection antibodies, were sequentially added to the wells. After incubation and thorough washing to remove unbound substances, the substrate solution containing tetramethylbenzidine (TMB) was added. Under the action of HRP, TMB was catalytically converted into a blue product, which turned yellow upon the addition of an acidic stop solution. The absorbance was measured at 450 nm using a microplate reader, and the hormone concentrations were calculated based on standard curves. The color intensity was positively correlated with the concentration of the corresponding hormone in the sample.

### 4.4. Real-Time Quantitative PCR Verification

Based on the research findings of Liu Hailun et al. (2023) [29], nineteen structural genes were selected for real-time quantitative PCR (RT-qPCR) analysis. Gene-specific primers were designed using Primer3 (https://primer3.ut.ee/, accessed on 10 August 2024) and synthesized by Shanghai Bioengineering Co., Ltd. Total RNA was extracted using the Steady Pure Quick RNA Extraction Kit (produced by ACCURATE BIOTECHNOLOGY, Hunan, China, https://agbio.com.cn/, accessed on 15 July 2024). The extracted RNA was subsequently used for cDNA synthesis with the Evo M-MLV RT Mix Kit with gDNA Clean for qPCR Ver.2 (AG11728) and SYBR^®^ Green Premix Pro Taq HS qPCR Kit (AG11701) from the same manufacturer. The qRT-PCR was conducted with the qTOWER3 QPCR system (Analytik Jena AG, Jena, Germany). The relative gene expression levels were calculated using the 2^−ΔΔCt^ method, with litchi actin as the internal reference gene. The details of the structural gene primers are provided in Table 1.

### 4.5. Statistical Analysis Methods

Statistical analysis was performed using SAS 9.4 software. A *t*-test was used to assess the significance of differences between T and CK at the same sampling time. Pearson’s correlation analysis was performed using the PROC CORR procedure. The Shapiro–Wilk test was used to assess the normality of variables involved in the Pearson correlation analysis. ** indicates a highly significant difference at *p* < 0.01; * indicates a significant difference at *p* < 0.05; and error bars represent the standard error.

## 5. Conclusions

Foliar calcium–magnesium nutrition treatment upregulated the expression of the five key *LcCDPK* gene family members *LcCDPK1*, *LcCDPK4*, *LcCDPK5*, *LcCDPK9*, and *LcCDPK17* through increasing the pulp ABA content, which led to increasing CDPK levels. Additionally, T increased Ca^2+^ and Mg^2+^ concentrations, which further activated and enhanced CDPK activity. Through these two synergistic regulatory pathways, T promoted the synthesis activity of sucrose-metabolizing enzymes, which facilitated the accumulation of sucrose and other soluble sugars, leading it to ultimately overcome the “sugar receding” phenomenon of ‘Feizixiao’ litchi pulp. In practical production, foliar calcium–magnesium nutrition can be applied as a cultivation strategy to mitigate this issue. Furthermore, the findings of this study provide a theoretical basis for further research on litchi fruit quality regulation and innovating cultivation techniques.

## Figures and Tables

**Table 1:** Structural genes and actin primers.

Primer Name	Gene ID	Sequence (5′-3′)	Primer Name	Gene ID	Sequence (5′-3′)
Actin_F	DQ990337.1	TTGGATTCTGGTGATGGTGTG	*LcCDPK10_F*	*LITCHI015851*	GGAACTCTGGACTGTGACGA
Actin_R	DQ990337.1	CAGCAAGGTCCAACCGAAG	*LcCDPK10_R*	*LITCHI015851*	CATCGTTGGGGCTGGAAATT
*LcCDPK1_F*	*LITCHI001674*	GAGGAGGAGGAGGAGGAAGA	*LcCDPK11_F*	*LITCHI019005*	GTGTGGAGAAAGGGAGTGGA
*LcCDPK1_R*	*LITCHI001674*	TCTTGGCTTTGGGGATGACT	*LcCDPK11_R*	*LITCHI019005*	ATCGAATAGCTCCCCACCTG
*LcCDPK2_F*	*LITCHI002195*	TTTGATGCGGTGTTGAAGGG	*LcCDPK12_F*	*LITCHI019021*	ATGTGGCCCCAGAAGTGTTA
*LcCDPK2_R*	*LITCHI002195*	TCTGTCAGGAGCAACACCAT	*LcCDPK12_R*	*LITCHI019021*	GTCAACCGCTTCTTGGGATC
*LcCDPK3_F*	*LITCHI006474*	CAAGGCCAATGGAGATCGTG	*LcCDPK13_F*	*LITCHI022010*	AAAACGAGCATCTGGAGCAC
*LcCDPK3_R*	*LITCHI006474*	AGCAATTCACCTCCCTCACA	*LcCDPK13_R*	*LITCHI022010*	GCGCAAATTCCCCGAAAATG
*LcCDPK4_F*	*LITCHI006812*	ACATGCTCGTTGTGCCTATG	*LcCDPK14_F*	*LITCHI022230*	CACACACGCATCACTGACAA
*LcCDPK4_R*	*LITCHI006812*	TTTGCAAGGACTCGGCTAGA	*LcCDPK14_R*	*LITCHI022230*	TATCATCACCTCACGTCGCA
*LcCDPK5_F*	*LITCHI007334*	GGCAAGAAACTGGGTCAAGG	*LcCDPK15_F*	*LITCHI023733*	TCGGTAGAGGGCAATTTGGT
*LcCDPK5_R*	*LITCHI007334*	ACTCTCCTCCCTCACAAAGC	*LcCDPK15_R*	*LITCHI023733*	CGCACAACTCCATCACCAAA
*LcCDPK6_F*	*LITCHI008906*	GCGGAATTATGGCCCAGAAG	*LcCDPK16_F*	*LITCHI023923*	ATGGACACTGGCAAGAGAGG
*LcCDPK6_R*	*LITCHI008906*	GGGATCAGGGTCAAGCATCT	*LcCDPK16_R*	*LITCHI023923*	GGTGAACTGAAACCGCAACA
*LcCDPK7_F*	*LITCHI010062*	CAACATGCACCAAAACAGCC	*LcCDPK17_F*	*LITCHI027028*	ACTTGGTGTTGGAGTTGTGC
*LcCDPK7_R*	*LITCHI010062*	TTCCCAGTTGCCTTCTCAGT	*LcCDPK17_R*	*LITCHI027028*	CTCCGGCTTCAAGTCCCTAT
*LcCDPK8_F*	*LITCHI011639*	GTGGTGCAGATCAAAGGGAC	*LcCDPK18_F*	*LITCHI027050*	ATTGTTGGGGTTGTGGAAGC
*LcCDPK8_R*	*LITCHI011639*	ATGCATGACCCCAAGAGAGT	*LcCDPK18_R*	*LITCHI027050*	GCTGCCAACCACATCAGAAA
*LcCDPK9_F*	*LITCHI014137*	CAACAGGGAGGAAGTTTGCC	*LcCDPK19_F*	*LITCHI029637*	GCTCCTGAAGTATTGCGTCG
*LcCDPK9_R*	*LITCHI014137*	CCAGCACACAACTCCATCAC	*LcCDPK19_R*	*LITCHI029637*	TTCCTGACTAGCTCCTTGGC

## Data Availability

Data are contained within the article.

## Abbreviations

The following abbreviations are used in this manuscript:

CDPK	calcium-dependent protein kinases
Ca^2+^	water-soluble calcium
Mg^2+^	water-soluble magnesium
CK	control group
T	treatment group
DAA	days after anthesis
ABA	abscisic acid
EMP-TCA	Embden–Meyerhof–Parnas-tricarboxylic acid
PPP	pentose phosphate pathway
CP	cytochrome respiration pathway
AP	cyanide-resistant respiratory pathways
HK	hexokinase
Ser/Thr	serine/threonine
CAM	calmodulin-like
SPS	sucrose-phosphate synthase
*h*	hue angle
HPLC	high-performance liquid chromatography
ICP-OES	inductively coupled plasma optical emission spectrometry
BR	brassinosteroids
SA	salicylic acid
IAA	auxins
GA_3_	gibberellins
MeJA	methyl jasmonate
ELISA	enzyme-linked immunosorbent assay
PBS	phosphate-buffered saline
SS	sucrose synthase
SOX	sorbitol oxidase
RT-qPCR	real-time quantitative PCR
